# Protective Effect of Polymethoxyflavones Isolated from *Kaempferia parviflora* against TNF-α-Induced Human Dermal Fibroblast Damage

**DOI:** 10.3390/antiox10101609

**Published:** 2021-10-13

**Authors:** Hung Manh Phung, Sullim Lee, Sukyung Hong, Sojung Lee, Kiwon Jung, Ki Sung Kang

**Affiliations:** 1 College of Korean Medicine, Gachon University, Seongnam 13120, Korea; manhspkt92@gmail.com; 2 Department of Life Science, College of Bio-Nano Technology, Gachon University, Seongnam 13120, Korea; sullimlee@gachon.ac.kr; 3 College of Pharmacy, C.H.A University, Seongnam 13488, Korea; hongsk@chauniv.ac.kr (S.H.); sojung@chauniv.ac.kr (S.L.)

**Keywords:** skin aging, *Kaempferia parviflora*, 5,7,4′ trimethoxyflavone, human dermal fibroblasts, tumor necrosis factor-α, reactive oxygen species, inflammation

## Abstract

Similar to other organs, the skin undergoes a natural aging process. Moreover, constant direct exposure to environmental stresses, including ultraviolet irradiation, causes the signs of skin aging to appear rather early. Reactive oxygen species (ROS) and inflammatory responses accelerate skin damage in extrinsic aging. In this study, we aimed to investigate the skin protective effects of polymethoxyflavones found in *Kaempferia parviflora* against oxidative stress and inflammation-induced damage in human dermal fibroblasts (HDFs) stimulated by tumor necrosis factor-α (TNF-α). The experimental data identified 5,7,4′ trimethoxyflavone (TMF) as the most potent constituent in preventing TNF-α-induced HDF damage among the tested compounds and it was not only effective in inhibiting matrix metalloproteinase-1 (MMP-1) production but also in stimulating collagen, type I, and alpha 1 (COLIA1) expression. TMF suppressed TNF-α-stimulated generation of ROS and pro-inflammatory mediators, such as cyclooxygenase-2 (COX-2), interleukin (IL)-1β, and IL-6 in HDFs. TMF also inhibited the pathways regulating fibroblast damage, including mitogen-activated protein kinase (MAPK), activator protein 1 (AP-1), and nuclear factor-kappa B (NF-κB). In conclusion, TMF may be a potential agent for preventing skin aging and other dermatological disorders associated with oxidative stress and inflammation.

## 1. Introduction

Aging is a natural process involving gradual changes that begin in early adulthood. Many bodily functions begin to deteriorate gradually during the early middle age, including skin structure and function. The skin is a true reflection of the changes that accumulate over time. The natural aging process (intrinsic aging) normally begins in the mid-twenties and is characterized by fine wrinkles as well as thin and transparent, sagging, and dry skin. Although these changes usually begin in the twenties, the signs of intrinsic aging are typically not visible for decades. However, recently, the signs of skin aging tend to appear earlier with more severe symptoms, such as deep wrinkles or leathery skin, as the skin is frequently exposed to extreme environmental factors, including ultraviolet (UV) radiation, smoking, pollutants, and toxins (extrinsic aging) [1]. These environment stresses are considered culprit-induced degradation of the extracellular matrix (ECM), which results in wrinkle formation.

The ECM, including elastins, collagen fibrils, and ground substance, is mainly synthesized by dermal fibroblasts located in the dermal layer of the skin, which maintains the structural integrity of most tissues [2]. Hence, fibroblast dysfunction has an impact on the mechanical properties of skin connective tissue, which is associated with the formation of wrinkles [3]. In addition, Borg et al. [4] showed that the degradation of ECM components such as collagen is critically controlled by cytokines and inflammatory mediators [4].

TNF-α, a pro-inflammatory cytokine secreted from keratinocytes and fibroblasts following UV, has a vital part in mediating aging and inflammatory diseases in the skin. In terms of cutaneous aging, TNF-α regulates various signaling pathway-induced production cases of collagenase as well as inhibites collagen bio-synthesis in dermal fibroblasts [4]. In detail, TNF-α triggers ROS and pro-inflammatory cytokine production, and the activation of both activator protein 1 (AP-1) and nuclear factor-kappa B (NF-κB), which lead to the generation of interstitial collagenase (MMP-1) and the degradation of collagen fibrils [4,5,6].

Additionally, TNF-α is also considered an important mediator in inflammatory skin disorders, including psoriasis, a popular chronic inflammatory ailment marked by aberrant keratinocyte proliferation, increased cutaneous vascularity, and numerous infiltrating inflammatory cells. TNF-α provoke the production of pro-inflammatory cytokines associated with psoriatic pathogenesis, such as IL-1 and IL-6. IL-1 promotes the generation of additional cytokines as well as the expression of cytokeratin 6 (CK6), a hallmark of hyperproliferative and activated keratinocytes, while IL-6 causes T-cell proliferation and keratinocyte hyperproliferation [5,6]. Furthermore, TNF-α works in part by enhancing the elevated level of active, phosphorylated NF-κB, a key transcription factor implicated in the development of psoriasis [7].

Thus, modulation of TNF-α activity could be a potent approach for the development of new therapeutic agents in preventing skin aging and inflammatory skin diseases. Natural products represent a diverse and abundant source of antioxidant and anti-inflammation agents that are not only effective in preventing signs of age but are also safe and less irritating to the skin. Therefore, natural plants and herbs have been used in traditional practices to preserve youthfulness of the skin. For example, the Naxi people, an ethnic group that lives in Southwest China, traditionally apply an oil prepared from the seed of *Pandanus utilis* and the root of *Lithospermum erythrorhizon* to prevent skin aging [8].

Ginger (*Zingiber officinale*) has long been used as a food seasoning or as a medicinal agent for treatment [9]. It has been used for centuries in Europe and the Middle East, mainly in Asia and India, to treat disorders such as arthritis, abdominal pain, asthma, and diabetes [10]. In addition to the efficacy of ginger, the pharmacological effects of the *Kaempferia parviflora* (KP) rhizome, also called black ginger, which has been traditionally used in Thailand and Laos in recent years, has been explored recently. *K. parviflora* belongs to the ginger family (Zingiberaceae) and is cultivated mainly in Thailand, Vietnam, and Laos [11,12]. The black ginger plant has dark-purple roots [13] and unlike general ginger, is a perennial herb that grows to a height of approximately 90 cm in the dark [14]. Although they share similar morphological characteristics, their compositions differ. In particular, rhizomes have been used as folk medicine for centuries as nutritional supplements and hypoglycemic agents, and to improve circulatory and digestive functions [13]. In traditional Thai medicine, KP powder is consumed as a decoction with alcohol. Nowadays, it is ingested in a variety of ways, such as through tablets or black ginger honey.

**Figure 1:**
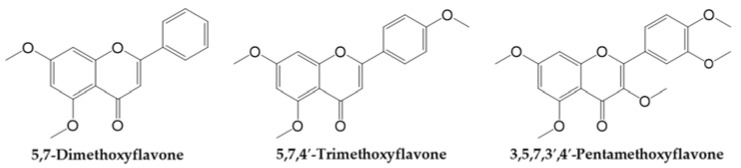
Chemical structure of polymethoxyflavones in *Kaempferia parviflora* (KP), including 5,7-Dimethoxyflavone (**DMF**), 5,7,4′-Trimethoxyflavone (**TMF**), and 3,5,7,3′,4′-Pentamethoxyflavone (**PMF**).

The rhizome of black ginger is a key part of the plant and contains several active ingredients. It contains 28 flavonoids such as polymethoxyflavones, including 3,5,7,3′,4′-pentamethoxyflavone (PMF), 5,7,4′ trimethoxyflavone (TMF), and 5,7-dimethoxyflavone (DMF) (Figure 1), which are detected at high concentrations in black ginger and are reported to have many health benefits such as improved blood flow and antioxidant, anti-inflammatory, and anti-allergic properties, as well as demonstrate to improve gastric ulcers [13,14,15,16,17,18]. Black ginger plants are cultivated in various places and both the properties and the efficacy of the rhizome may vary depending on environmental factors, such as the climate of the area of cultivation. In particular, DMF, TMF, and PMF are the main constituents of black ginger [13,19,20] and are mainly effective in improving sexual function and treating erectile dysfunction. TMF has strong vasodilation and anti-inflammation, anti-fungal, anti-mycobacterial, and anti-malarial activities [9,17,19]. In addition, TMF and DMF exert anti-degranulation and anti-inflammatory effects in RBL–2H3 cells. This indicates that the polymethoxyflavones found in the rhizome prevents vascular inflammation [21]. DMF mainly has anti-cancer and vasodilator effects on the liver, oral cavity, esophagus, and lungs. Moreover, DMF has been shown to attenuate obesity and metabolic syndromes. DMF inhibited the accumulation of triglycerides in 3T3-L1 adipocytes and displayed anti-obesity effects in obese C57BL/6J mice [22]. Considering the various therapeutic benefits of black ginger reported in studies thus far, black ginger represents a potential substance that may be effective in the treatment of various diseases.

With regard to the protective effects on skin, black ginger, together with its main constituents, has been shown to be a potential antioxidant source, which protects the skin against UVB-induced oxidative stress. Park et al. [23] reported that an ethanol extract of KP enhances the expression of catalase (an antioxidant enzyme) in UVB-stimulated skin of hairless mice. In addition, a previous study indicated that DMF, another antioxidant present in black ginger, suppresses UVB-stimulated ROS production in Hs68 human dermal fibroblasts [24]. However, there has been limited research on the efficacy of the two other polymethoxyflavones in KP, namely TMF and PMF, which share the flavone backbone of DMF (Figure 1) in the prevention of skin damage. From the known skin protective effect of DMF, as well as its chemical structural similarity to TMF and PMF, we sought to determine whether the chemical structure of these compounds collates with the skin protective effects.

In this study, we aimed to investigate protective effects of DMF, TMF, and PMF from KP against TNF-α-induced HDF damage via expression of MMP-1 and collagen type I alpha 1 (COLIA1), and other relevant signaling pathways.

## 2. Materials and Methods

### 2.1. Extraction Methods

KP obtained from the Gyeongdong market (Seoul, Korea) was ground to a powder form using a blender (HR2607/00, Philips, Amsterdam, the Netherlands). A sample of the powder (50 g) was extracted with 500 mL of ethanol solvent at room temperature for 2 h and filtered before drying in a rotary evaporator (N-1110, Eyela, NY, USA). After evaporation of the solvent, the extract was dried overnight in a vacuum oven (OV-11/12, Jeiotech, Seoul, Korea). The KP extract (0.1 g) was dissolved in 1 mL of methanol to constitute a 0.1 g/mL stock solution.

### 2.2. High-Performance Liquid Chromatography (HPLC) Analysis

HPLC (Agilent 1260 Infinity II, CA, USA) analysis was performed. Compounds were separated on an Aegispak C_18_ column (250 × 4.6 mm, 5 μm particle). Standard samples (0.001 g; DMF, TMF, and PMF) were dissolved in 1 mL of methanol to make a 0.001 g/mL stock solution. All the samples were filtered using a 0.45 μm filter (FNY-422-013, Biofil, India) before injection into the HPLC system.

The conditions of HPLC analysis were as follows: column temperature, 50 °C; injection volume, 10 μL. The mobile phase consisted of 0.1% phosphoric acid aqueous solution (solvent A) and 0.1% phosphoric acid in acetonitrile (solvent B) at 1 mL/min flow rate. The gradient elution program used for the sample was as follows: 30–40% of solvent A from 0–5 min; 40–43% from 5–20 min; 43–80% from 20–21 min; 80% from 21–30 min; 80–30% from 30–31 min; and 30% from 31–40 min. The UV detection wavelength was 254 nm.

The content was calculated using the area of the peak in the HPLC analysis. The formula used to calculate the content was as follows: content (g/100) = (sample area/STD area) × (sample dilution volume × dilution factor/STD dilution volume × dilution factor) × (STD amount taken/sample amount taken) × 100.

### 2.3. Cell Culture and Drug Preparation

Human dermal fibroblasts (HDFs) juvenile foreskin (PromoCell GmbH, Sickingenstr, Heidelberg, Germany) were grown in Dulbecco’s modified Eagle’s medium (DMEM; Corning, Manassas, VA, USA) containing 10% fetal bovine serum (FBS; Atlas, Fort Collins, CO, USA) and 100 U/mL of penicillin–streptomycin (Gibco, Grand Island, NY, USA) in a humid atmosphere containing 5% CO_2_ at 37 °C. 5,7-Dimethoxyflavone (DMF, Chengdu Biopurify Phytochemicals Ltd., Chengdu, Sichuan, China), 5,7,4′-Trimethoxyflavone (TMF, Chengdu Biopurify Phytochemicals Ltd.), 3,5,7,3′,4′-Pentamethoxyflavone (PMF, Chengdu Biopurify Phytochemicals Ltd.), and Quercetin (Sigma, St. Louis, MO, USA) were dissolved in dimethyl sulfoxide (DMSO; Sigma-Aldrich, St. Louis, MO, USA) to produce stock solutions at a concentration of 100 mM. TNF-α (PeproTech, Rocky Hill, NJ, USA) stock solution (20 µg/mL) was prepared in 1% filtered bovine serum albumin (BSA, Sigma-Aldrich). The final concentration of DMSO was maintained under 1‰, in which the cytotoxicity of the vehicle (DMSO) could not be detected compared to the control cells. For cell experiments, 24 h after plating the cells, HDFs were starved in serum-free DMEM overnight and treated with TNF-α and other compounds.

### 2.4. Cell Viability

HDFs were plated in 96-well plates (1 × 10^4^ cells/well) and allowed to adhere for 24 h. After overnight starvation in serum-free medium, the cells were treated with a range of concentrations of samples and incubated for 24 h. Next, 10 μL of EZ-Cytox solution (Dogen, Seoul, Korea) was added to each well, incubated for 30 min, and OD450 was measured using a microplate reader (SPARK 10M; Tecan, Männedorf, Switzerland). The viability of the HDFs was assessed using the following formula:

Cell viability (%) = (OD450 sample—OD450 blank)/(OD450 control—OD450 blank) × 100.

### 2.5. Intracellular ROS Assay

HDFs were plated in 96-well plates (1 × 10^4^ cells/well) and incubated for 24 h. The cells were starved overnight in serum-free DMEM. Following this, the HDFs were exposed to 6.25 and 12.5 µM TMF for 1 h and subsequently with a mixture of 20 ng/mL TNF-α and 10 µM DCFDA (Sigma-Aldrich) for 15 min. After washing with phosphate-buffered saline (PBS; Welgene, Gyeongsangbuk, Korea), the fluorescence intensity of DCFDA was analyzed using a microplate reader (SPARK 10M; Tecan) at excitation and emission wavelengths of 485 nm and 535 nm, respectively. Fluorescent images were captured using an IX51 fluorescence microscope (Olympus, Tokyo, Japan) equipped with a CCD camera.

### 2.6. Quantitative Real-Time Polymerase Chain Reaction (qRT-PCR)

HDFs were plated in 6-well plates at a density of 3 × 10^5^ cells/well. After starving in serum-free DMEM overnight, the cells were treated with specific doses of samples for 1 h and subsequently exposed to 20 ng/mL TNF-α for 4 h (to check mRNA expression of IL-1β and IL-6) and 24 h (to investigate MMP-1 and COLIA1 mRNA expression). Total cellular RNA was isolated using the RNeasy Mini Kit (Qiagen, Germantown, MD, USA). RNA was reverse-transcribed into cDNA using the RevertAid First Strand cDNA Synthesis kit (Thermo Fisher Scientific, Eugene, OR 97402, USA). PCR was performed using the AccuPower^®^ 2X GreenStar™ qPCR Master Mix (Bioneer, Daejeon, Korea) using sense and antisense primers as listed in Table 1. β-actin was chosen as the housekeeping gene. The amplification conditions were as follows: 95 °C for 5 min; followed by 40 cycles of 95 °C for 15 s; 60 °C for 30 s; and 95 °C for 30 s for all primers using the Quant Studio 3 real-time PCR system (Applied Biosystems, Foster City, CA, USA).

### 2.7. Enzyme-Linked Immunosorbent Assay (ELISA)

HDFs were plated in 48-well plates (2 × 10^4^ cells/well) and incubated for 24 h. Next, the medium was discarded, replaced by serum-free DMEM, and the plates were incubated overnight. The cells were then treated with the indicated concentrations of the samples for 1 h and subsequently with 20 ng/mL TNF-α for 12 h (to test the secretion of IL-1β and IL-6) and 24 h (to investigate MMP-1 and COLIA1 secretion). The concentration of IL-1β, IL-6, MMP-1, and COLIA1 in the supernatant were determined using sandwich ELISA kits (R&D Systems, Minneapolis, MN, USA).

### 2.8. Western Blotting

HDFs were plated in 60 mm culture dishes at a density of 4 × 10^5^ cells/dish and starved overnight with serum-free DMEM. Next, the cells were treated with 6.25 and 12.5 μM TMF for 1 h and then with 20 ng/mL TNF-α for 15 min (for the detection of the protein expression of ERK, p-ERK, p38, p-p38, c-Jun, p-c-Jun, c-Fos, p-c-Fos, p65, and p-p65). To assess p65 translocation and COX-2 expression, the cells were incubated with 20 ng/mL TNF-α for 4 and 6 h, respectively. Next, the cells were washed with PBS and lysed in 1 × radioimmunoprecipitation assay (RIPA) buffer supplemented with 1× protease inhibitor cocktail (Roche Diagnostics, Indianapolis, IN, USA), 1 mM Na_3_VO_4_, and 5 mM NaF to obtain whole-cell lysates. In addition, the cells were washed with PBS and lysed in the cytoplasmic lysis buffer containing 10 mM KCl, 0.1 mM EDTA, 10 mM HEPES (pH 7.5), 1 mM DTT, 0.5% Nonidet−40, and 0.5 mM PMSF, along with the 1× protease inhibitor cocktail. After 10 min incubation, the pallets obtained after centrifuging were subsequently pipetted in nuclear extraction buffer containing 400 mM NaCl, 1 mM EDTA, 20 mM HEPES (pH 7.5), 1 mM DTT, and 1 mM PMSF with 1× protease inhibitor cocktail, and incubated in ice for 30 min. After centrifuging, the supernatants were collected and used as nuclear lysates. Protein concentration in the lysate was determined using the BCA Protein Assay Kit (Merck, Darmstadt, Germany). Equal amounts of protein samples were separated on a polyacrylamide gel and transferred to a polyvinylidene difluoride membrane (PVDF, Dogen, Seoul, Korea) activated by methanol. After blocking with 5% non-fat milk in tris-buffered saline containing 0.1% Tween-20 (TBS-T) for 1 h, the membranes were probed with the primary antibodies (Cell Signaling, Danvers, MA, USA) of interest diluted in 1% BSA overnight at 4 °C and with suitable secondary antibodies (Cell Signaling, Danvers, MA, USA) diluted in 1% BSA for 1 h. Next, the membranes were treated with the EZ–Western Lumi Femto Kit (Dogen, Seoul, Korea) and the immunoreactive bands were captured using the Fusion Solo Chemiluminescence System (PEQLAB Biotechnologie GmbH, Erlangen, Germany).

### 2.9. Immunofluorescence Staining

HDFs (4 × 10^4^ cells/well) were plated in an 8-well chamber slide and allowed to adhere for 24 h. Next, the medium in the chamber slide was replaced with serum-free DMEM. After 24 h of incubation, the cells were treated with 6.25 and 12.5 μM TMF for 1 h and subsequently with 20 ng/mL TNF-α for 4 h. The cells were fixed with 4% paraformaldehyde in PBS (T&I, Gangwon, Korea) for 10 min and blocked with 5% normal goat serum (R&D systems, Minneapolis, MN, USA) containing 0.3% Triton™ X-100 (Bio-Rad, Hercules, CA, USA) in PBS for 1 h. Next, the cells were probed with primary antibody (Cell Signaling, Danvers, MA, USA) at 4 °C overnight and with secondary antibodies conjugated with Alexa Fluor^®^ 488 (Cell Signaling) for 2 h. Subsequently, the cells were stained with 10 µM Hoechst (Sigma-Aldrich) and incubated in the dark for 10 min. Fluorescent images were obtained using an IX51 fluorescence microscope (Olympus) coupled to a CCD camera.

### 2.10. Statistical Methods

Experimental data are presented as mean ± standard error of mean (SEM). One-way analysis of variance (ANOVA) with Tukey’s honest significance test was applied to assess statistical differences. Statistical significance was set at *p* < 0.05.

## 3. Results

### 3.1. Polymethoxyflavones in KP

**Figure 2:**
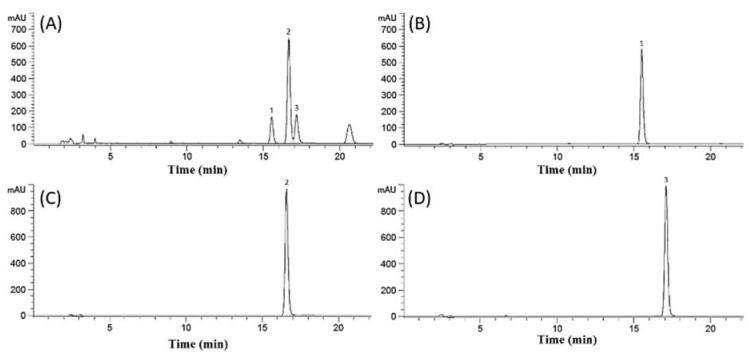
Chromatograms of *Kaempferia parviflora* extract (0.1 g/mL) and standard solutions (0.001 g/mL). (**A**) Extract of KP, (**B**) PMF, (**C**) DMF, and (**D**) TMF. Peaks: (1) PMF, (2) DMF, and (3) TMF. Abbreviations: KP, *Kaempferia parviflora*; DMF, 5,7-dimethoxyflavone; TMF, 5,7,4′-trimethoxyflavone; and PMF, 3,5,7,3′,4′-pentamethoxyflavone.

The amounts of PMF, DMF, and TMF in the KP extract was determined by HPLC analysis (Figure 2). Content analysis using HPLC revealed 85.05 ± 0.79, 283.42 ± 4.09, and 106.19 ± 2.95 mg/g of PMF, DMF, and TMF, respectively, in the KP extract.

### 3.2. Effect of KP-Derived Polymethoxyflavones on the Viability of HDFs

**Figure 3:**
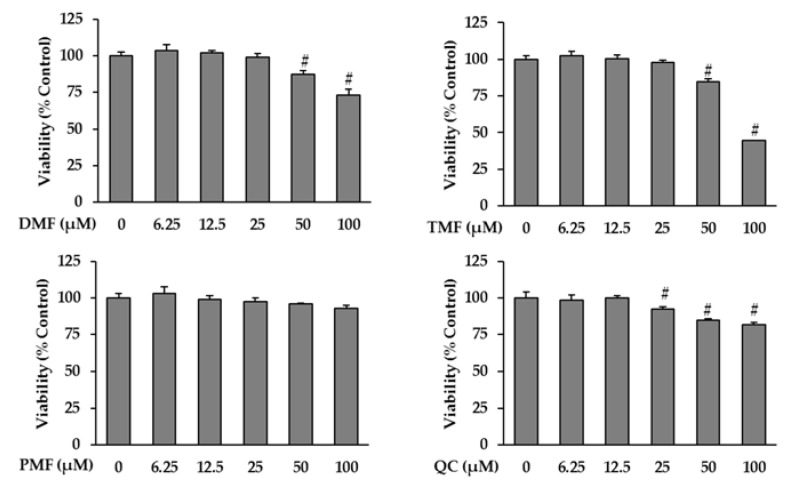
Effects of DMF, TMF, PMF, and QC (positive control) on cell viability in HDFs. HDFs were plated in 96-well plates at a density of 1 × 10^4^ cells/well and starved with serum-free media for 24 h. Next, the cells were treated with specific concentrations of the sample for 24 h. Cell viability was determined using the Ez-Cytox kit. The data are presented as mean ± SEM (*n* = 3). # *p* < 0.05 compared to non-treated group. Abbreviations: DMF, 5,7-dimethoxyflavone; TMF, 5,7,4′-trimethoxyflavone; PMF, 3,5,7,3′,4′-pentamethoxyflavone; and QC, Quercetin.

Prior to analyzing the anti-aging effects of KP-derived polymethoxyflavones, including DMF, TMF, and PMF, we investigated their cytotoxicity on HDFs to determine a suitable concentration range for further experiments. Quercetin, a strong antioxidant that exerts a protective effect on the skin as evidenced in previous anti-photoaging studies [25,26] and used in cosmetic formulations [27], was selected as a positive control. As shown in Figure 3, there was a significant decrease in the viability of cells treated with 50 and 100 µM DMF to 87.45 ± 1.49% (*p* = 0.05) and 73.02 ± 2.37% (*p* = 0.05), respectively. Similarly, exposure to 50 and 100 µM TMF reduced cell viability to 84.47 ± 1.14% (*p* = 0.05) and 44.65 ± 0.07% (*p* = 0.05), respectively. PMF did not show considerable cytotoxicity effects on HDFs, whereas the viability of HDFs treated with 25, 50, and 100 µM of Quercetin significantly reduced to 92.18 ± 0.87% (*p* = 0.05), 84.70 ± 0.47% (*p* = 0.05), and 81.75 ± 0.72% (*p* = 0.05), respectively. Hence, the maximum dose used for to subsequent experiments was set at 12.5 µM.

### 3.3. Effect of DMF, TMF, and PMF on MMP-1 Expression in TNF-α-Stimulated HDFs

**Figure 4:**
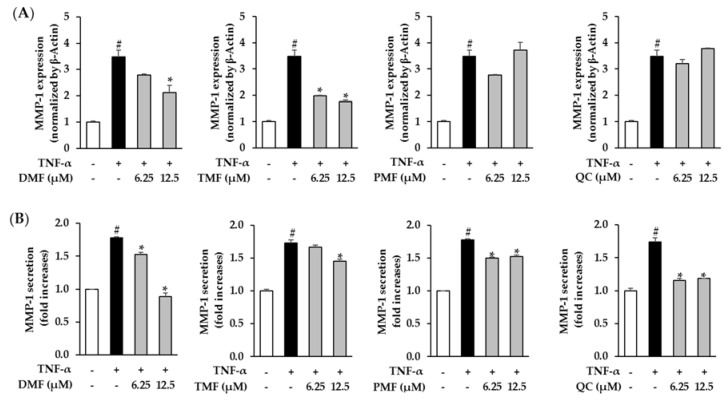
Effects of DMF, TMF, PMF, and QC (positive control) on MMP-1 mRNA expression (**A**) and secretion (**B**). HDFs were plated at a density of 2 × 10^4^ cells/well in a 48-well plate and starved with serum-free media for 24 h. Next, the cells were treated with specific concentrations of the samples for 1 h before exposure to 20 ng/mL TNF-α for 24 h. The MMP-1 mRNA level was assessed using qRT-PCR analysis. MMP-1 secretion in the cell supernatants was determined using an ELISA kit. The data were expressed as mean ± standard error of mean (SEM) (*n* = 2). # *p* < 0.05 compared to non-treated group and * *p* < 0.05 compared to TNF-α-treated group. Abbreviations: DMF, 5,7-dimethoxyflavone; TMF, 5,7,4′-trimethoxyflavone; PMF, 3,5,7,3′,4′-pentamethoxyflavone; and QC, Quercetin.

Next, we analyzed the skin protective effects of DMF, TMF, PMF, and Quercetin by verifying the mRNA expression and secretion of MMP-1 induced by TNF-α in HDFs. Treatment with 20 ng/mL TNF-α significantly stimulated mRNA expression of MMP-1 by 3.48 ± 0.25-folds (*p* = 0.05) compared to the control group. Among the tested samples, TMF exerted the strongest inhibitory effect on mRNA expression of MMP-1. Treatment with 6.25 and 12.5 µM TMF diminished mRNA expression of MMP-1 to 1.98 ± 0.03-folds (*p* = 0.05) and 1.76 ± 0.06-folds (*p* = 0.05), respectively, while DMF considerably mitigated mRNA expression of MMP-1 to 2.10 ± 0.29-folds (*p* = 0.05) at the dose of 12.5 µM. PMF and Quercetin did not significantly inhibit the effect on mRNA expression of MMP-1 in TNF-α-stimulated HDFs (Figure 4A). In terms of MMP-1 secretion, exposure to 20 ng/mL TNF-α promoted MMP-1 secretion to 1.76 ± 0.01-folds (*p* = 0.05), whereas treatment with most of the samples significantly decreased MMP-1 secretion in a concentration-dependent manner. Among the studied samples, the compound most effective in inhibiting MMP-1 secretion was DMF when it attenuated the secretion of MMP-1 to 1.53 ± 0.03-folds (*p* = 0.05) and 0.88 ± 0.06-folds (*p* = 0.05) at the concentration of 6.25 and 12.5 µM, respectively. In addition, exposure to 12.5 µM TMF 3 clearly reduced the secretion of MMP-1 by 1.45 ± 0.03-folds (*p* = 0.05) and treatment of cells with 6.25 and 12.5 µM PMF mitigated MMP-1 secretion by 1.50 ± 0.01-folds (*p* = 0.05) and 1.52 ± 0.02-folds (*p* = 0.05), respectively. Quercetin (used as a positive control) also suppressed MMP-1 secretion by 1.16 ± 0.03 (*p* = 0.05) and 1.19 ± 0.05-folds (*p* = 0.05) at concentrations of 6.25 and 12.5 µM, respectively (Figure 4B).

### 3.4. Effect of DMF, TMF, and PMF on COLIA1 Expression in TNF-α-Stimulated HDFs

**Figure 5:**
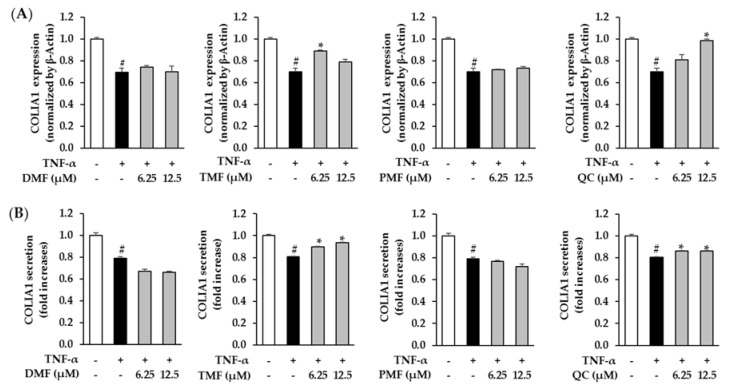
Effects of DMF, TMF, PMF, and QC (positive control) on COLIA1 mRNA expression (**A**) and secretion (**B**). HDFs were plated in a 48-well plate at a density of 2 × 10^4^ cells/well and starved with serum-free media for 24 h. Next, the cells were treated with specific concentrations of the samples for 1 h before exposure to 20 ng/mL TNF-α for 24 h. COLIA1 mRNA was assessed using qRT-PCR analysis. COLIA1 secretion in the cell supernatants was determined using an ELISA kit. The data are presented as mean ± SEM (*n* = 2). # *p* < 0.05 compared to non-treated group and * *p* < 0.05 compared to TNF-α-treated group. Abbreviations: DMF, 5,7–dimethoxyflavone; TMF, 5,7,4′-trimethoxyflavone; PMF, 3,5,7,3′,4′-pentamethoxyflavone; and QC, Quercetin.

Subsequently, the anti-aging effect of the compounds on the skin was further investigated by analyzing the mRNA expression and secretion of collagen, type I-alpha 1 (COLIA1). In the TNF-α-treated group, the mRNA expression and secretion of COLIA1 decreased to 0.70 ± 0.03-folds (*p* = 0.05) and 0.80 ± 0.00-folds (*p* = 0.05), respectively. Among the polymethoxyflavones isolated from KP, including DMF, TMF, and PMF, only TMF showed a positive effect in restoring the COLIA1 level. TMF significantly restored the mRNA expression of COLIA1 to 0.89 ± 0.01-folds (*p* = 0.05) at the concentration of 6.25 µM and enhanced COLIA1 secretion to 0.90 ± 0.00-folds (*p* = 0.05) and 0.94 ± 0.00-folds (*p* = 0.05) at the concentrations of 6.25 and 12.5 µM, respectively. Exposure to 12.5 µM Quercetin increased mRNA expression and secretion of COLIA1 to 0.99 ± 0.01-folds (*p* = 0.05) and 0.86 ± 0.00-folds (*p* = 0.05), respectively (Figure 5).

**Figure 6:**
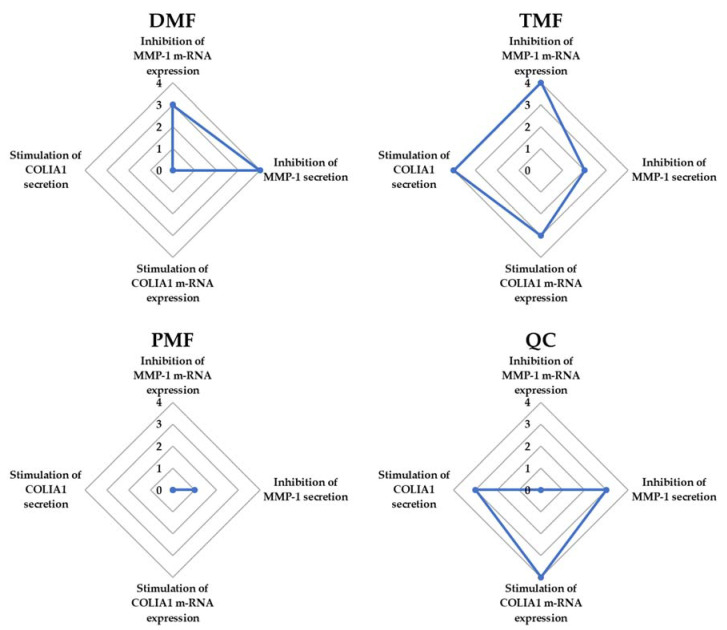
The spider chart for the efficiency comparison of DMF, TMF, PMF, and QC (positive control) on five markers associated with HDF damage, such as inhibition of MMP-1 mRNA expression, inhibition of MMP-1 secretion of MMP-1, stimulation of COLIA1 mRNA expression, and stimulation of COLIA1 secretion. The scores were defined from four, corresponding to the strongest effect, to 1, corresponding to the lowest effect, among the four tested samples, in addition to 0, corresponding to no effect on each factor. Abbreviations: DMF, 5,7-dimethoxyflavone; TMF, 5,7,4′-trimethoxyflavone; PMF, 3,5,7,3′,4′-pentamethoxyflavone; and QC, Quercetin.

The spider chart for the efficiency comparison of DMF, TMF, PMF, and Quercetin (positive control) on five factors associated with HDF damage including inhibition of MMP-1 mRNA expression, inhibition of MMP-1 secretion of MMP-1, stimulation of COLIA1 mRNA expression, and stimulation of COLIA1 secretion indicated that TMF showed the most potent compound in preventing TNF-α-induced fibroblast damage among the tested polymethoxyflavones (Figure 6). Therefore, we chose TMF for further experiments to identify the mechanism through which TMF exerts protective effects against TNF-α-induced HDF damage.

### 3.5. Effect of TMF on TNF-α-Induced Pro-Inflammatory Mediators in HDFs

**Figure 7:**
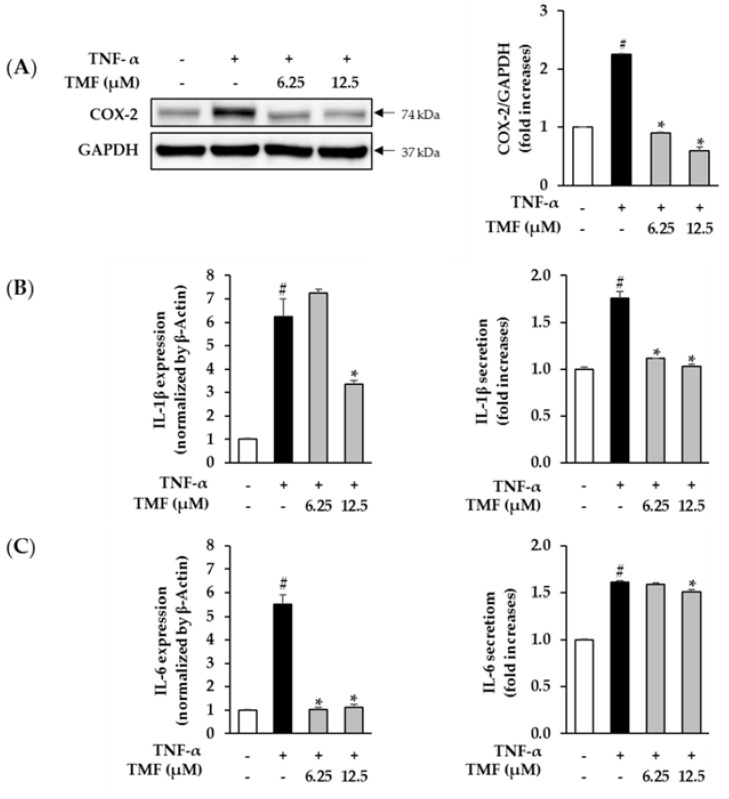
Effect of TMF on TNF-α-induced COX-2, IL-1β, and IL-6 expression in HDFs. (**A**) Expression of the pro-inflammatory mediator, COX-2, in TNF-α-stimulated HDFs was detected using western blotting. The data are described as mean ± SEM (*n* = 3). # *p* < 0.05 compared to non–treated group and * *p* < 0.05 compared to TNF-α–treated group. The mRNA expression and secretion of pro–inflammatory cytokines IL-1β (**B**) and IL-6 (**C**) were assessed using qRT-PCR analysis and the ELISA kit, respectively. The data are described as mean ± SEM (*n* = 2). # *p* < 0.05 compared to non-treated group and * *p* < 0.05 compared to TNF-α-treated group. Abbreviations: TMF, 5,7,4′-trimethoxyflavone, and TNF-α, tumor necrosis factor alpha.

Next, we investigated the effect of TMF on TNF-α-induced generation of pro-inflammatory regulators, including COX-2, IL-1β, and IL-6, in HDFs that are key factors in the skin-aging process. As shown in Figure 6, treatment with 20 ng/mL TNF-α significantly increased the expression of COX-2, IL-1β, and IL-6. However, the upregulation was reversed in the groups treated with TMF. Briefly, TMF reduced the expression of COX-2 in a dose-dependent manner compared to that in the TNF-α-treated group (Figure 7A). Regarding the expression of IL-1β, treatment with 20 ng/mL TNF-α enhanced its mRNA expression and secretion to 6.23 ± 0.76-folds (*p* = 0.05) and 1.76 ± 0.07-folds (*p* = 0.05), respectively. However, exposure to 12.5 µM TMF suppressed mRNA expression and secretion of IL-1β to 3.37 ± 0.13-folds (*p* = 0.05) and 1.03 ± 0.02-folds (*p* = 0.05), respectively (Figure 7B). In a similar manner, exposure to 20 ng/mL TNF-α stimulated mRNA expression and secretion of IL-6 to 5.53 ± 0.38-folds (*p* = 0.05) and 1.61 ± 0.01-folds (*p* = 0.05), respectively. However, treatment with 12.5 µM TMF significantly attenuated mRNA expression and secretion of IL-6 to 1.12 ± 0.13-folds (*p* = 0.05) and 1.51 ± 0.02-folds (*p* = 0.05), respectively (Figure 7C).

### 3.6. Effect of TMF on TNF-α-Induced ROS Production in HDFs

**Figure 8:**
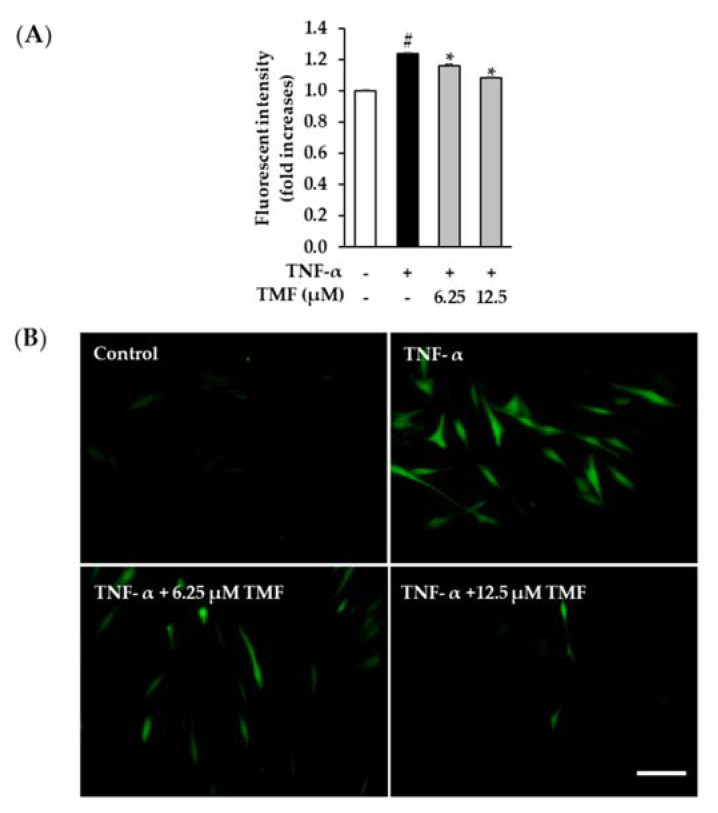
Inhibitory effect of TMF on ROS generation caused by TNF-α in NHDFs. (**A**) Briefly, 1 × 10^4^ cells were seeded in 96-well plates and incubated for 24 h. The cells were continuously starved in serum-free DMEM for 24 h. After that, the cells were exposed to 6.25 and 12.5 µM TMF for 1 h and subsequently with 20 ng/mL TNF-α and 10 µM DCFDA for 15 min. The DCFDA intensity was measured using a fluorescence microplate reader. (**B**) The fluorescent images of DCFDA-positive cells were captured by a fluorescence microscope (20× magnification, 100 µm scale bar). The data are depicted as mean ± SEM (*n* = 3). # *p* < 0.05 compared to non–treated group and * *p* < 0.05 compared to TNF-α-treated group. Abbreviations: TMF, 5,7,4′-trimethoxyflavone, and TNF-α, tumor necrosis factor alpha.

Besides the induction of inflammation responses, TNF-α has become known as an oxidative stress stimulator in dermal fibroblasts. Hence, we continuously assessed the effect of TMF on TNF-α-induced ROS production in HDFs. As shown in Figure 8A, treatment with 20 ng/mL TNF-α increased ROS production to 1.27 ± 0.01-folds (*p* = 0.05) and decreased by 1.07 ± 0.01-folds (*p* = 0.05) and 1.01 ± 0.01- folds (*p* = 0.05) in cells treated with 6.25 and 12.5 µM TMF, respectively. In addition, the fluorescent images clearly visualized the inhibitory effect of TMF in ROS formation when the DCFDA intensity significantly enhanced in the TNF-α-treated group and reduced in the groups exposed to TMF in a dose-dependent manner (Figure 8B).

### 3.7. Effect of TMF on TNF-α-Induced Phosphorylation of Mitogen-Activated Protein Kinase (MAPKs) in HDFs

**Figure 9:**
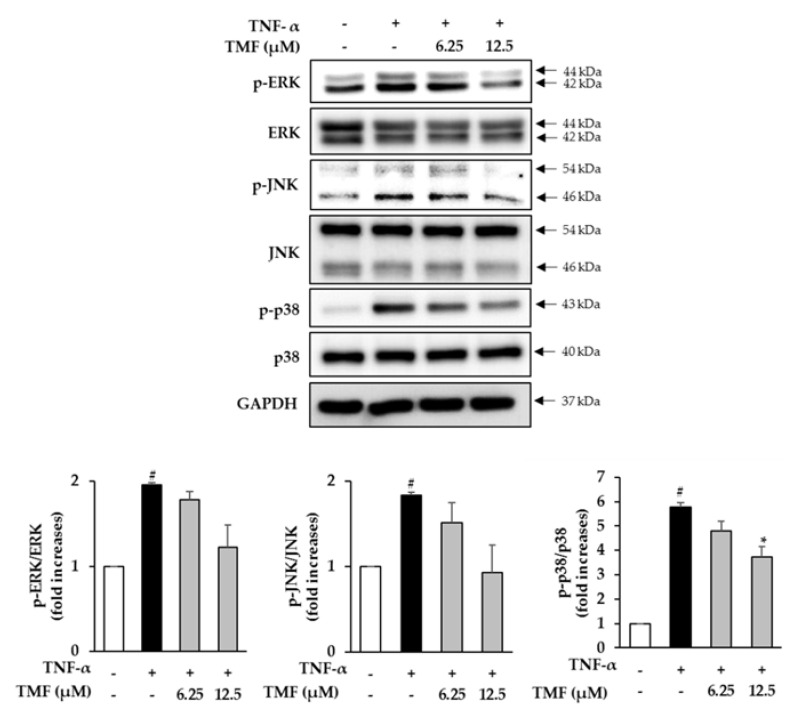
Effect of TMF on the phosphorylation of MAPKs in TNF-α-stimulated HDFs. Expression of p-ERK, ERK, p-JNK, JNK, p–p38, p38, and GAPDH was assessed using western blotting. The data are depicted as mean ± SEM (*n* = 3). # *p* < 0.05 compared to non-treated group and * *p* < 0.05 compared to TNF-α-treated group. Abbreviations: TMF, 5,7,4′-Trimethoxyflavone, and TNF-α, tumor necrosis factor alpha.

Next, we assessed the effect of TMF on the phosphorylation of MAPKs in TNF-α-stimulated HDFs. Treatment with 20 ng/mL TNF-α markedly promoted the phosphorylation of JNK, p38, and ERK in HDFs compared to that in the control cells, whereas treatment with TMF inhibited phosphorylation in a concentration-dependent manner (Figure 9).

### 3.8. Effect of TMF on the Expression and Phosphorylation of c-Fos and c-Jun in TNF-α-Stimulated HDFs

**Figure 10:**
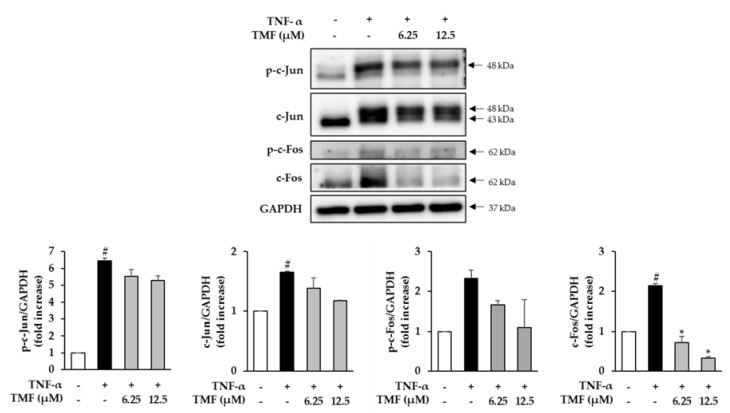
Effect of TMF on the expression and phosphorylation of c-Fos and c-Jun in TNF-α-stimulated HDFs. Expression of p-c-Jun, c-Jun, p-c-Fos, c-Fos, and GAPDH was assessed using western blotting. The data are presented as mean ± SEM (*n* = 3). # *p* < 0.05 compared to non–treated group and * *p* < 0.05 compared to TNF-α-treated group. Abbreviations: TMF, 5,7,4′-trimethoxyflavone, and TNF-α, tumor necrosis factor alpha.

We further investigated the effect of TMF on the expression of c-Fos and c-Jun, the two subunits of AP-1, in TNF-α-stimulated HDFs. Treatment with 20 ng/mL TNF-α markedly promoted both phosphorylation and expression of c-Jun and c-Fos in the HDFs compared to that in the untreated group. However, the TNF-α stimulated upregulation was suppressed in the TMF-treated groups (Figure 10).

### 3.9. Effect of TMF on TNF-α-Induced Phosphorylation and Translocation of NF-κB in HDFs

**Figure 11:**
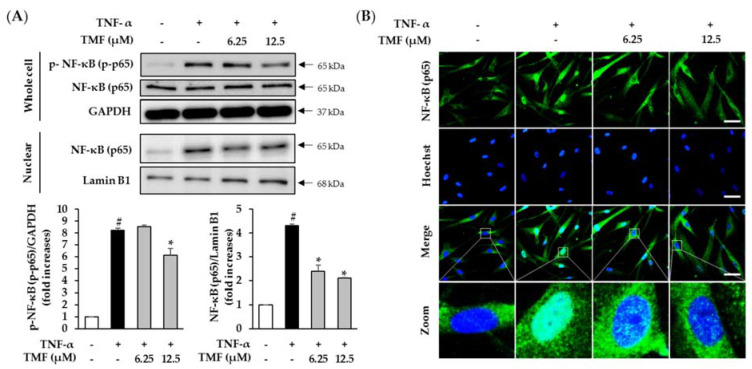
Effect of TMF on TNF–α–induced phosphorylation and translocation of NF-κB in HDFs. (**A**) Expression and phosphorylation of NF–κB (p65) was determined by immunoblotting. The data are expressed as mean ± SEM (*n* = 3). # *p* < 0.05 compared to non-treated group and * *p* < 0.05 compared to TNF-α-treated group. (**B**) Translocation of NF-κB (p65) from the cytoplasm to nuclear was visualized using immunofluorescence staining (40× magnification, 100 µm scale bar). Abbreviations: TMF, 5,7,4′-trimethoxyflavone, and TNF-α, tumor necrosis factor alpha.

Ultimately, we analyzed the effect of TMF on TNF-α-induced phosphorylation and translocation of NF-κB in HDFs. As shown in Figure 11, the phosphorylation and translocation of NF-κB was significantly increased in cells treated with 20 ng/mL TNF-α and inhibited in cells treated with TMF in a concentration-dependent manner.

## 4. Discussion

Skin, the human body’s biggest organ, protects the interior organs by functioning as a barrier from chemical and physical contaminants. Extracellular matrix (ECM) proteins are produced by resident fibroblasts in the dermis and are responsible for the skin’s resilience and strength. As a result of the disorganization of the ECM in the dermis, the skin begins to age. Although there are other kinds of collagen in the dermal ECM (III, V, and VII), Type I collagen is the most significant structural protein in the dermal ECM. The major cause of aged skin, which appears thin, smooth, dry, and lacks elasticity, is diminished collagen fibrils and decreased Type I collagen production. During skin aging, MMP-1, an interstitial collagenase, is responsible for the destruction of collagen fibrils. Varani et al. found that as age increases, MMP-1 levels rise and collagen expression falls in human skin; moreover, excessive ECM breakdown in the dermis by MMP-1 contributes significantly to connective tissue inflammation. As a result, in skin aging, the balance between MMP 1 and Type I collagen expression is critical [28,29].

Skin may age either intrinsically, via the chronological aging process that affects all bodily organs, or extrinsically, as a result of environmental stresses such as UV radiation, cigarette smoke, stress, and pollution. Both extrinsic and intrinsic skin aging processes are linked to skin inflammation via the generation of inflammatory cytokines. During intrinsic and extrinsic aging, different skin cells produce TNF-α, a 17-kDa inflammatory cytokine that functions as an important modulator of the metabolic response and cell activities. UV irradiation stimulates TNF-α receptors on the cell surface, which is one of the most important causes of skin aging. TNF-α reduces collagen expression and increases MMP expression, resulting in ECM deposition. Apart from the context of skin aging, TNF-α is also considered a vital regulator in inflammatory skin diseases such as psoriasis. TNF-α increases the production of pro-inflammatory cytokines including IL-1 and IL-6, and activates the NF-κB-signaling pathway, which promotes the development of psoriasis [5,6,7]. Thus, modulation of TNF-α activity could be a potent approach for the development of new therapeutic agents in preventing skin aging and inflammatory skin disorders [28,29].

In this study, we investigated the protective effects of polymethoxyflavones from the *Kaempferia parviflora* (KP) rhizome including polymethoxyflavones such as 3,5,7,3′,4′-pentamethoxyflavone (PMF), 5,7,4′ trimethoxyflavone (TMF), and 5,7-dimethoxyflavone (DMF) against TNF-α-induced human dermal fibroblast (HDF) damage via expression of MMP-1 and COLIA1. The experimental data indicated that the dose-dependent manner could not be observed in the effect of PMF and quercetin in inhibiting mRNA expression and secretion of MMP-1, and neither in the effect of TMF in stimulating COLIA1 mRNA expression. Additionally, among the tested samples, TMF was the most potent constituent compound of KP that prevented fibroblast damage induced by TNF-α, wherein it not only clearly inhibited MMP-1 expression but also stimulated COLIA1 expression. The skin protective effect of TMF was stronger than that of quercetin. Subsequently, a series of mechanistic assessments were performed to determine the skin protective efficacy of this compound.

Pro-inflammatory mediators such as COX-2, IL-1, and IL-6 play a key role in promoting the signs of cutaneous aging and inflammatory skin conditions. In skin aging, IL-6 is a regulator correlated with the generation of wrinkles on the skin, whereas COX-2 is well known to mediate the synthesis of prostaglandin E2 (PGE2), a lipid-derived signaling molecule involved in the production of MMP-1 and the suppression of collagen biosynthesis in HDFs [30]. In psoriasis, IL-1 stimulates the synthesis of additional cytokines and the expression of cytokeratin 6 (CK6), a marker of hyperproliferative and activated keratinocytes, whereas IL-6 stimulates T-cell proliferation and keratinocyte hyperproliferation [5,6]. Luteolin, a flavonoid found in vegetables, herbs, spices, and flowers, mitigated the photoaging progress in the epidermis and dermis layer by suppressing ROS and various pro-inflammatory mediators (e.g., TNF-α, IL-1β, IL-8, IL-6, IL-22, IL-17, and COX-2) [31]. In a similar manner, TMF also successfully inhibited the expression of IL-1β, IL-6, and COX-2 caused by TNF-α, consolidating its potential skin protective effects associated with anti-inflammatory activities.

Apart from inflammation responses, oxidative stress is a major factor associated with the aging process of the skin. ROS arising from both intrinsic (chronological aging) and extrinsic aging (photoaging) stimulate the expression of MMPs and suppress transforming growth factor beta (TGF-β) signaling, which causes collagen breakdown and inhibition of collagen fibril biosynthesis [32]. Thus, in the cosmetic industry, building an antioxidant shield for the skin from natural sources is one of the main approaches in the development of skin care products [33]. A recent study by Li et al. indicated that orange cold-pressed oil, a rich polymethoxyflavone source, exerted preventive effects against UVB-induced oxidative damage in mouse skin [34]. In a similar manner, in this study, TMF also showed a strong inhibitory effect on TNF-α-induced ROS production in HDFs. This pharmacological effect may be related to the position and number of methoxyl groups (-OCH3) in the chemical structure of polymethoxyflavones associated with antioxidant properties, as they increase the electronic density of the aromatic ring [35].

MAPK, together with two downstream pathways, namely AP-1 and NF-κB, associated with oxidative stress and inflammatory responses, play a central role in regulating skin damage [36,37,38]. ROS and inflammatory cytokines activate MAPKs, inducing an increase in the expression of c-Jun and c-Fos in the nucleus. These subunits undergo heterodimerization to produce activated AP-1 complexes. In the epidermis and dermis, AP-1 triggers the expression of MMP-1, MMP-9, and MMP-3, which degrade collagen and other components of the skin ECM [37]. In addition, activation of MAPKs also induces phosphorylation and translocation of NF-κB, which leads to the production of MMP-1 and pro-inflammatory cytokines [36,39]. Thus, reversal of these pathways plays an important role in delaying the aging progress of the skin. Previous reports have indicated that treatment with MAPK, AP-1, and NF-κB inhibitors reversed the photoaging process [37,39]. In this study, TMF also emerged as a potential MAPK, AP-1, and NF-κB-suppressor. HDFs treated with TMF showed attenuated phosphorylation of MAPKs, reduced expression and phosphorylation of c-Fos and c-Jun, and decreased phosphorylation and translocation of NF-κB, all of which resulted in the inhibition of MMP-1 production and collagen degradation.

In summary, among the polymethoxyflavones found in KP, TMF constitutes the most potent compound in preventing TNF-α-induced HDF damage by not only inhibiting MMP-1 expression but also by restoring COLIA1 expression. The skin protective mechanism of TMF in TNF-α-stimulated in HDFs is mediated through the suppression of oxidative stress and inflammatory response via inhibition of ROS and pro-inflammatory mediators, including COX-2, IL-1β, and IL-6 production. Moreover, TMF also mitigates phosphorylation of MAPKs, phosphorylation and translocation of NF-κB, as well as the expression and phosphorylation of c-Fos and c-Jun, which inhibit MMP-1 generation and breakdown of the collagen fibrils downstream.

Nonetheless, there may be some possible limitations in this study. For example, the potential of TMF in preventing skin aging was only studied in HDFs. To fully elucidate the anti-skin aging effect of TMF, the study should be extended to investigating on many different cell lines including keratinocytes, melanocytes, and organotypic 3D skin models. In addition, the protective mechanisms of polymethoxyflavones against TNF-α-induced senescence in dermal fibroblasts have not been presented in this study, although it should be considered in upcoming studies [40]. Furthermore, apart from skin-aging, ROS is also considered an important factor in promoting carcinogenesis [41]. Thus, the antioxidants from KP, including DMF, TMP, and PMF, may have anti-skin cancer effects and this aspect should be studied in the future.

## 5. Conclusions

The results of this study indicate that among the major bioactive compounds found in KP, TMF represents the most potent constituent in preventing TNF-α-induced HDF damage, where it exerts a dual efficacy in inhibiting MMP-1 production and upregulating COLIA1 expression in TNF-α-stimulated fibroblasts. The protective effect of TMF may be due to the suppression of ROS and pro-inflammatory regulators, such as COX-2, IL-1β, and IL-6 at upstream, that lead to the reversal of the signal transduction pathways associated with MMP-1 and pro-inflammatory cytokine production, as well as with collagen degradation at downstream, including MAPK, AP-1, and NF-κB pathways. Collectively, our data suggest that TMF may be a potential antioxidant and anti-inflammatory agent in preventing skin aging and other dermatological disorders associated with oxidative stress and inflammation. In addition, to fully elucidate the anti-skin aging effect of TMF, further studies on the skin protective efficacy of TMF on many different types of skin cells, such as keratinocytes, melanocytes, and organotypic 3D skin models, must be conducted.

## Figures and Tables

**Table 1:** Primer sequences.

Gene	Sense Primer Sequence (5′–3′)	Antisense Primer Sequence (5′–3′)
MMP-1	ATTCTACTGATATCGGGGCTTT	ATGTCCTTGGGGTATCCGTGTA
COLIA1	CTCGAGGTGGACACCACCCT	CAGCTGGATGGCCACATCGG
IL-1β	CTGTCCTGCGTGTTGAAAGA	TTCTGCTTGAGAGGTGCTGA-3
IL-6	CTCCTTCTCCACAAGCGCC	GCCGAAGAGCCCTCAGGC
β-actin	AGAGATGGCCACGGCTGCTT	ATTTGCGGTGGACGATGGAG

Abbreviations: MMP-1, matrix metalloproteinase-1; COLIA1, collagen; type I, alpha 1; IL–1β, Interleukin-1β; and IL-6, Interleukin-6.

## Data Availability

Data is contained within the article.
